# Supplementation of protease to low amino acid diets containing superdose level of phytase for wean-to-finish pigs: effects on performance, postweaning intestinal health and carcass characteristics

**DOI:** 10.1093/tas/txab088

**Published:** 2021-05-16

**Authors:** J Y Perez-Palencia, R S Samuel, C L Levesque

**Affiliations:** Department of Animal Science, South Dakota State University, Brookings, SD 57007, USA

**Keywords:** enzymes, fecal score, intestinal health, intestinal permeability

## Abstract

This experiment investigated the effects of protease supplementation to low amino acid (AA) diets containing phytase on pig growth performance, postweaning intestinal health and carcass characteristics. A total of 936 weaned pigs (21 d of age, initial BW 5.87 ± 0.31 kg) were used in a 2 × 2 factorial design comparing the main effects of AA supply [standard feeding program: balanced for all nutrients with adjustment of Ca and P due to inclusion of phytase (2,500 FTU/kg in Phase 1 to 4; 500 FTU/kg in Phase 5 to 9) vs. low AA feeding program: 15% lower standardized ileal digestible lysine with relative reduction of all other essential AA] and protease level (0 vs. 0.0125%). Pens were assigned to dietary treatment according to a randomized complete block design with 26 pigs per pen and nine replicates per dietary treatment. Feed and water were provided on an ad libitum basis for all phases throughout the wean-to-finish period. Feed intake and body weight were determined every 2 wk during nursery period and monthly in the grow-finish period. Intestinal health in the first 17 d was assessed based on lactulose:mannitol ratio (L:M), serum IgA, and pen diarrhea assessment. Overall, pigs fed standard wean-to-finish diets had greater (*P* < 0.05) ADG and G:F than pigs fed low AA diets. Pig growth performance was not different throughout the wean-to-finish period with or without protease supplementation and with no interaction between AA supply and protease supplementation. There were no differences among dietary treatments for carcass characteristics. No difference was observed for urinary L:M and serum IgA; however, the L:M ratio was approximately 32% lower in pigs fed low AA diets + protease compared with pigs fed standard and low AA diets at d 5 and d 17 postweaning. Pigs fed protease supplemented diets had lower incidence of diarrhea (*χ*^2^ < 0.05) compared with pigs fed diets without protease. Results of the experiment indicate that dietary protease supplementation benefits intestinal health of nursery pigs.

## INTRODUCTION

In the past few decades, feed enzymes have been one of the most prominent biotechnological enhancements in monogastric nutrition (Brameld and Parr, 2016). This nutritional strategy has led to improved nutrient utilization, reduced feed cost, and reductions in manure nitrogen (N) and inorganic phosphorus (P) content (Kim et al., 2020). Phytase is the most common feed enzyme used in the swine industry; its inclusion in the diet aims to reduce the antinutritional effect of phytate while improving, primarily, P and calcium (Ca) and, secondarily, amino acids (AA) and energy digestibility (Jang et al., 2017; She et al, 2017). Consequently, phytase allows adjustments around the inclusion of inorganic macromineral sources in diet formulations. High levels of added phytase (superdosing) have been observed to enhance nutrient digestibility and pig growth performance and have been observed to be more effective during the nursery phase than the grow-finish phase (Holloway et al., 2019). Proteases, another commonly used enzyme in monogastric diets, may increase the rate of hydrolysis of protein sources, increasing AA availability and reducing N excretion. Collectively, data from several studies evaluating the effects of protease supplementation in pig diets report positive impacts on nutrient digestibility (Lee et al., 2018). However, results related to pig growth performance are inconsistent (Zamora et al., 2011; Mc Alpine et al., 2012; Zuo et al., 2015; Tactacan et al., 2016; Upadhaya et al., 2016; Chen et al., 2017; Lei et al., 2017).

Dietary enzyme supplementation has also been associated with positive impacts on gut health under specific production conditions (Kiarie et al., 2013; Zuo et al., 2015; Tactacan et al., 2016; Duarte et al., 2019). In swine production, the stressful events associated with weaning have negative effects on gut health and the overall growth performance of young pigs (McLamb et al., 2013; Khafipour et al., 2014). High-quality protein sources are commonly used in nursery pig diets in an attempt to compensate for a lesser capacity to digest dietary nutrients during this critical period after weaning (Berrocoso et al., 2012; Berrocoso et al., 2013). Otherwise, undigested protein (i.e., N) can increase the intestinal protein fermentation, which in turn has been related to postweaning diarrhea and growth of potentially pathogenic bacteria (Rist et al., 2013; Wu et al., 2015). In this context, protease supplementation may contribute to improving dietary protein utilization, promoting intestinal functionality and health status of nursery pigs. However, to our knowledge, this hypothesis has not been addressed in experiments with pigs kept under commercial conditions.

Other little-known aspects about dietary supplementation of protease in swine diets include the potential interactive effects when combined with other enzymes such as phytase and its effects on growth performance or intestinal health. Therefore, the objective of this study was to evaluate the effects of protease supplementation to low AA diets containing phytase throughout the wean-to-finish period on pig growth performance, postweaning intestinal health, and carcass characteristics under commercial conditions. We hypothesized that dietary protease supplementation increases AA digestibility to the point of offsetting the 15% reduction in AA content in the diet for wean-to-finish pigs. In addition, protease supplementation improves gut health of nursery pigs by reducing indigestible N and consequently the proliferation of pathogenic bacteria under commercial conditions.

## MATERIAL AND METHODS

The experimental protocols used in this study were approved by the South Dakota State University Institutional Animal Care and Use Committee (IACUC #18-093A). The experiment was conducted in the commercial wean-to-finish barn at South Dakota State University (SDSU), in Brookings, SD 57006, USA.

### Animals and Housing

One group of approximately 1,200 newly weaned pigs were randomly allotted to pens at the SDSU off-site wean-to-finish barn upon arrival; any injured, sick, or small pigs were separated and housed in “off-test” pens. Thereafter, a total of 936 weaned pigs (offspring of PIC females and PIC Duroc-280 boars; equal barrows and gilts per pen, 21 d of age) with initial body weight (BW) of 5.87 ± 0.31 kg were used in a 2 × 2 factorial design (two dietary AA levels × two protease levels) and assigned to one of the four dietary treatments with nine replicate pens of 26 pigs (3.1 m × 6.9 m; approximately 0.82 m^2^ per pig). All pens contained one five space dry feeder (178 cm total length; SD Industries, Alexandria, SD 57311) and two cup waterers for ad libitum access to feed and water. The facility is equipped with a single M-Series FEEDPro system (Feedlogic by ComDel Innovation, Willmar, MN 56201) for feeding which was used to monitor feed dispensed to each pen, according to the assigned treatment. The barn operated on mechanical ventilation, with temperature setpoints at 26.1, 23.3, 24.4, 22.7, 20.5, 18.3, 16.7, and 16.1 ºC for d 1, 15, 29, 43, 57, 85, 113, and 134, respectively.

Throughout the trial, daily animal monitoring included records of veterinary treatment on a per pen basis including number of pigs/pen, drug administered, dosage, duration, reason for pig removal (i.e., dead, untreatable health issue such as umbilical prolapse, morbundity), and incidence of health concerns (e.g., diarrhea, coughing). Water medications for the entire barn (R-Pen Penicillin G Soluble Antibiotic, Alpharma LLC, 1399 Bridgewater, NJ 08807, USA) were provided according to the directions of the attending veterinarian between d 2 and d 7 of entry to barn. Individual spot treatments were used as the next line of defense for poor health, incidences of lameness, or other illness.

### Experimental Diets

Diet formulations consisted of a nine-phase wean-to-finish feeding program containing phytase (2,500 FTU/kg in Phase 1 to 4 and 500 FTU/kg in Phase 5 to 9; standard) and a reduced AA feeding program (standard feeding program with 15% lower standardized ileal digestible (SID) lysine and relative reduction of all other essential AA; low AA). Protease was included at 0 or 0.0125% (Jefo Protease, Jefo Nutrition Inc., Saint-Hyacinthe, QC, Canada) at the expense of corn in standard and low AA diets to create standard + protease and low AA + protease diets. All diets were formulated to meet or exceed the NRC (2012) recommended requirements for pigs with adjustment of the inclusion of P due to the expected uplift of phytase, which received a credit of 0.15% P release regardless of dietary inclusion level throughout all diets and any Ca or other digestibility credit.

During the nursery phase, pigs were fed the assigned experimental diets in a four-phase feeding program. All pigs received a common Phase 1 diet according to a feed budget of 0.9 kg/pig (ME: 3418 kcal/kg; SID Lys: 1.40%; standardized total tract digestible P – STTD P: 0.40%; Ca: 0.76%) and experimental diets according to a feed budget of 3.4, 4.5, and 24 kg/pig in Phases 2, 3, and 4, respectively (Table 1). During the grow-finish period, a five-phase feeding program was used and followed PIC (2016) recommended feed budgets/phase. The five-phase feeding program corresponded to the following live weight ranges (kg): 23–41; 41–59; 59–82; 82–104; 104-market for Phases 5 to 9, respectively. Control diets were formulated to meet NRC (2012) nutritional recommendations for net energy (NE) and SID Lys, while low AA diets were 15% lower SID lysine and relative reduction of all other essential AA. The PIC (2016) nutritional recommendations were followed for all other nutrient levels (Table 2).

**Table 1.** Experimental diets (as-fed basis)—nursery^*a*^

	Phase 2		Phase 3		Phase 4	
Ingredient, %	Standard	Low AA	Standard	Low AA	Standard	Low AA
Corn, yellow dent	44.32	49.21	50.44	52.22	56.71	58.67
Soybean meal	16.77	16.86	18.53	18.87	23.53	22.15
Soy protein product^*b*^	5.72	1.88	2.00	1.20	0.00	0.00
Soybean oil	0.78	0.44	0.60	0.47	0.49	0.45
Spray dried whey	23.86	24.00	12.10	12.10	0.00	0.00
Enzymatically treated soybean meal^*c*^	4.27	3.54	2.00	1.20	0.00	0.00
Distillers dried grains with solubles	0.00	0.00	10.00	10.00	15.00	15.00
l-Lysine HCl	0.39	0.28	0.58	0.38	0.66	0.45
dl-Methionine	0.19	0.11	0.17	0.08	0.17	0.07
l-Threonine	0.08	0.02	0.14	0.05	0.19	0.09
l-Valine	0.05	0.00	0.10	0.00	0.12	0.00
l-Tryptophan	0.01	0.00	0.04	0.01	0.05	0.02
Monocalcium phosphate, 21% P	2.14	2.13	1.37	1.36	0.99	1.00
Limestone	0.54	0.59	1.02	1.05	1.21	1.22
Salt	0.39	0.44	0.55	0.57	0.63	0.63
Vitamin premix^*d*^	0.05	0.05	0.05	0.05	0.05	0.05
Trace mineral premix^*e*^	0.15	0.15	0.15	0.15	0.15	0.15
Phytase^*f*^	0.05	0.05	0.05	0.05	0.05	0.05
Zinc oxide, 72% Zn	0.25	0.25	0.12	0.12	0.00	0.00
*Calculated composition*						
ME, kcal/kg	3,266	3,241	3,288	3,282	3,306	3,297
CP, %	21.7	19.2	20.7	19.5	21.0	20.0
SID Lys, %	1.40	1.19	1.35	1.15	1.33	1.13
SID Met, %	0.48	0.37	0.46	0.36	0.46	0.36
SID Thr, %	0.84	0.69	0.79	0.67	0.79	0.67
SID Trp, %	0.25	0.21	0.24	0.21	0.24	0.20
SID Val, %	0.96	0.81	0.92	0.78	0.90	0.77
STTD P, %	0.45	0.45	0.32	0.32	0.27	0.27
Ca, %	0.85	0.85	0.79	0.79	0.70	0.70

^*a*^Standard: Wean-to-finish feeding program formulated to meet or exceed the NRC (2012) recommended requirements for nursery pigs, including superdose level of phytase (2,500 FTU/kg); low AA: standard diet with a 15% reduction in SID AA levels. Protease was included at 0.0125% at the expense of corn in standard and low AA diets to create standard + protease and low AA + protease diets (Jefo Protease, Jefo Nutrition Inc., Saint-Hyacinthe, Qc, Canada).

^*b*^HP300 (56.71% CP), HAMLET PROTEIN Inc., 5289 Hamlet Drive, Findlay, OH 45840, USA.

^*c*^ARDEX, ADM, 77 West Wacker Drive, Suite 4600, Chicago, IL 60601, USA.

^*d*^J & R Distributing Inc. 518 Main Ave, Lake Norden, SD 57248, USA. Minimum provided per kg of diet: calcium 55 mg, vitamin A 11,000 IU, vitamin D3 1,650 IU, vitamin E 55 IU; vitamin B12 0.044 mg, menadione 4.4 mg, biotin 0.165 mg, folic acid 1.1 mg, niacin 55 mg, d-pantothenic acid 60.5 mg, vitamin B16 3.3 mg, riboflavin mg, 9.9 thiamine 3.3 mg.

^*e*^J & R Distributing Inc. 518 Main Ave, Lake Norden, SD 57248, USA. Minimum provided per kg of diet: copper 11 g, manganese 29.4 g, selenium 0.2 g, zinc 110 g.

^*f*^Quantum Blue (5,000 FTU/g), AB Vista, 150 South Pine Island Road (Suite 270), Plantation, FL 33324, USA. Provided 2,500 FTU/kg diet.

**Table 2.** Experimental diets (as-fed basis)—growing-finishing^*a*^

	Phase 5		Phase 6^*b*^		Phase 7		Phase 8		Phase 9	
Feeds	Standard	Low AA	Standard	Low AA	Standard	Low AA	Standard	Low AA	Standard	Low AA
Corn, yellow dent	56.62	63.25	62.37	67.57	68.51	73.60	73.06	77.62	78.59	82.76
Soybean meal	20.00	13.60	16.00	10.55	12.00	7.50	8.65	4.60	8.40	4.70
Soybean oil	0.90	0.30	0.70	0.30	0.70	0.20	0.75	0.30	0.72	0.30
Distillers dried grains with solubles	19.15	19.50	17.50	18.25	16.00	16.00	15.00	15.00	10.00	10.00
L-Lysine HCl	0.52	0.49	0.46	0.43	0.42	0.40	0.40	0.38	0.36	0.34
dl-Methionine	0.10	0.05	0.05	0.00	0.04	0.00	0.04	0.00	0.04	0.00
l-Threonine	0.13	0.11	0.11	0.08	0.09	0.07	0.08	0.07	0.07	0.06
l-Tryptophan	0.04	0.04	0.03	0.03	0.03	0.03	0.03	0.03	0.03	0.03
l-Valine	0.00	0.00	0.00	0.00	0.02	0.00	0.00	0.00	0.00	0.00
Monocalcium phosphate, 21%	0.57	0.68	0.79	0.82	0.35	0.39	0.22	0.25	0.09	0.12
Limestone	1.33	1.33	1.35	1.33	1.17	1.15	1.10	1.08	1.00	0.99
Salt	0.43	0.44	0.43	0.44	0.46	0.46	0.47	0.47	0.50	0.51
Vitamin premix^*c*^	0.05	0.05	0.05	0.05	0.05	0.05	0.05	0.05	0.05	0.05
Trace mineral premix^*d*^	0.15	0.15	0.15	0.15	0.15	0.15	0.15	0.15	0.15	0.15
Phytase^*e*^	0.01	0.01	0.01	0.01	0.01	0.01	0.01	0.01	0.01	0.01
*Calculated composition*										
ME, kcal/kg	3,344	3,314	3,328	3,310	3,352	3,328	3,364	3,343	3,368	3,348
CP, %	20.1	17.6	18.13	16.08	16.28	14.47	14.74	13.13	13.65	12.17
SID Lys, %	1.15	0.97	1.00	0.84	0.86	0.73	0.76	0.65	0.70	0.60
SID Met, %	0.39	0.32	0.32	0.25	0.29	0.23	0.27	0.21	0.25	0.20
SID Thr, %	0.70	0.60	0.62	0.52	0.54	0.46	0.48	0.41	0.44	0.38
SID Trp, %	0.21	0.18	0.18	0.15	0.16	0.13	0.14	0.12	0.13	0.11
SID Val, %	0.76	0.66	0.69	0.61	0.64	0.54	0.55	0.49	0.51	0.45
STTD P, %	0.26	0.27	0.29	0.29	0.20	0.20	0.17	0.17	0.13	0.14
Ca, %	0.68	0.68	0.71	0.69	0.56	0.55	0.50	0.49	0.45	0.44

^*a*^Standard: Wean-to-finish feeding program formulated to meet or exceed the NRC (2012) recommended requirements for growing pigs, including phytase (500 FTU/kg); low AA: standard diet with a 15% reduction in SID AA levels. Protease was included at 0.0125% at the expense of corn in standard and low AA diets to create standard + protease and low AA + protease diets (Jefo Protease, Jefo Nutrition Inc., Saint-Hyacinthe (Qc), Canada).

^*b*^Phase 6: due to a shortage of phytase, level was adjusted down slightly based on what was actually available. This occurred in the last batch of Phase 6 only.

^*c*^J & R Distributing Inc., 518 Main Ave, Lake Norden, SD 57248, USA. Minimum provided per kg of diet: calcium 55 mg, vitamin A 11,000 IU, vitamin D3 1,650 IU, vitamin E 55 IU; vitamin B12 0.044 mg, menadione 4.4 mg, biotin 0.165 mg, folic acid 1.1 mg, niacin 55 mg, d-pantothenic acid 60.5 mg, vitamin B16 3.3 mg, riboflavin mg, 9.9 thiamine 3.3 mg.

^*d*^J & R Distributing Inc., 518 Main Ave, Lake Norden, SD 57248, USA. Minimum provided per kg of diet: copper 11 g, manganese 29.4 g, selenium 0.2 g, zinc 110 g.

^*e*^AB Vista, 150 South Pine Island Road (Suite 270), Plantation, FL 33324, USA. Provided 500 FTU/kg diet.

### Growth Performance and Carcass Characteristics

In the nursery period, feed intake and BW were determined every other week for calculation of average daily gain (ADG), average daily feed (ADFI), and gain-to-feed ratio (G:F). During the grow-finish period these variables were measured monthly. A density stick was used to estimate feed in feeders by volume based on the previously determined equation:


$$\begin{array}{l} FL\;=\;-15.335*\left(X\right)+\;618.26 \end{array}$$

where FL = leftover in the feeder and X = the measurement of empty space in the feeders (inches).

The pigs were marketed over 5 wk with pigs selected for shipping based on visual identification by a trained staff person, starting on d 138 (wk 1: 144 pigs, wk 2: 262 pigs, wk 3: 253 pigs, wk 4: 130 pigs, wk 5: 127 pigs). Selected pigs were tattooed by treatment for identification at the commercial abattoir (Wholestone Farms, 900 S Platte Ave, Fremont, NE 68025, USA) where carcass data were collected (hot carcass weight, loin depth, and backfat thickness) from the first four groups of marketed pigs. One research personnel was at the slaughter plant at the time of processing to follow carcasses through the plant and collect the data. Hot carcass weight was collected following the standard protocol of the abbatoir and loin depth (mm) and backfat thickness (mm) were collected every other carcass using Fat-O-Meter (MPI-CG, Meat Probes, Inc. Topeka, KS, USA). This probe was introduced perpendicularly into the left side of the carcass at point P2 (i.e., 6 cm lateral to the carcass dorsal midline immediately caudal to the last rib). Percentage of carcass fat-free lean (FFL, %) was calculated using National Pork Producers Council equations (NPPC; 2000).

### Intestinal Health Measurements

On d 7, 10, and 14, fecal consistency of the pen was assessed visually using a fecal scoring scale with four categories (Pedersen and Toft, 2011). The four consistency categories were: score one = firm and shaped, score two = soft and shaped, score three = loose and score four = watery, where scores of 1 and 2 represented normal feces and scores of 3 and 4 represented diarrhea. For each pen, a single observer assigned the relative proportion of visible feces that fell within each category, as well as an overall pen score.

On d 5 and 17, a blood sample was collected from one average pig/pen from all pens assigned to standard, low AA, and low AA + protease diets for analysis of serum IgA (*n* = 9/dietary treatment). These treatments were chosen to determine potential effect of the protease on the immune response compared with the standard and low AA groups. Plasma was collected by centrifugation (2,000 × *g*, 15 min, 4°C), allocated into 1.5 mL microcentrifuge tubes, and stored at −20 °C until analysis (CR412, Jouan Inc., 170 Marcel Drive Winchester, VA 22602, USA). The differential sugar absorption test (DSAT) was completed over 3 d to coincide with blood collection (d 4 to 6 and d 16 to 18) using the same pigs as for blood sample collection. On each day of the DSAT test, equal numbers of pigs from each treatment were randomly transferred to one of nine individual crates (0.56 × 0.64 × 0.89 m^2^) with access to feed and water. A bolus that contained 5% of both lactulose and mannitol was orally administrated to the pigs at 15 mL/kg (Nguyen et al., 2014) using a syringe plus a fluid feeder probe followed by 6-h total urine collection. Thereafter, pigs were transferred back to their original pen. A urine subsample was collected after homogenization and stored at −80 °C for later determination of gut permeability by the lactulose:mannitol (L:M) ratio in urine (Hong et al., 2020).

### Chemical Analysis

Total concentrations of IgA in the serum of pigs was measured according to the method described by Chaytor et al. (2011) using commercially available ELISA kits (Bethyl Laboratories, Inc., Montgomery, TX 77356, USA). Each sample was analyzed in duplicate. The optical density (OD) value was read at 450 nm within 30 min by an ELISA plate reader (SpectraMAX190, Molecular Devices. 1311 Orleans Drive, Sunnyvale, CA 94089, USA). A standard curve of OD value versus IgA concentration was generated and the serum IgA concentration was then determined according to the standard curve.

Concentrations of lactulose and mannitol in urine samples were determined using a commercially available Intestinal Permeability Assay Kit (BioAssay Systems, 3191 Corporate Place, Hayward, CA 94545, USA) according to the manufacturer’s instructions.

### Statistical Analysis

The UNIVARIATE procedure of SAS (SAS Inst., Inc., Cary, NC, USA) was used to confirm the homogeneity of variance and to analyze for outliers. Performance, carcass measures, and treatment rate data were analyzed as a 2 × 2 factorial design using the PROC MIXED procedure in SAS, while IgA and DSAT data were analyzed as a randomized complete block design. In the model, the main effects of dietary AA supply, protease inclusion and their interactions were tested considering BW as the blocking factor and the pen as experimental unit. Least squares means were calculated for each independent variable. If main effects were significant at *P* ≤ 0.05, Tukey’s adjusted means test was used to detect differences among dietary treatments. For the variables “days to market” and “fecal scores”, data were analyzed as main effects of AA supply and protease inclusion levels using the PROC FREQ procedure in SAS. Variability in data was expressed as standard error of means (SEM).

## RESULTS

The analyzed chemical composition of experimental diets used in the present study corresponded to the targets in the diet formulations and were within the tolerance of normal variance (Table 3).

**Table 3.** Experimental diets (as-fed basis)—analyzed composition^*a*^

Item			ME, kcal/kg^*b*^	DM, %	CP, %	Lys, %	Thr, %	Met, %	Trp, %	Val, %	Calcium, %	Phos., %
**Phase 2**	*Standard*	Control	3,266	90.50	20.80	1.38	0.89	0.45	0.29	1.01	0.65	0.64
		Protease	3,266	89.70	21.40	1.45	0.93	0.47	0.28	1.00	0.72	0.73
	*Low AA*	Control	3,241	89.80	19.00	1.26	0.79	0.39	0.26	0.93	0.62	0.69
		Protease	3,241	89.50	19.00	1.27	0.79	0.38	0.26	0.91	0.76	0.75
**Phase 3**	*Standard*	Control	3,288	89.20	19.90	1.49	0.90	0.45	0.28	1.03	0.69	0.62
		Protease	3,288	89.40	20.50	1.43	0.89	0.41	0.28	1.02	0.72	0.58
	*Low AA*	Control	3,282	89.40	19.60	1.27	0.79	0.36	0.30	0.93	0.68	0.57
		Protease	3,282	88.90	19.50	1.32	0.80	0.35	0.31	0.90	0.70	0.61
**Phase 4**	*Standard*	Control	3,306	86.80	20.10	1.43	0.94	0.44	0.27	1.01	0.70	0.52
		Protease	3,306	86.50	20.40	1.45	0.90	0.44	0.27	1.02	0.84	0.61
	*Low AA*	Control	3,297	86.50	19.00	1.27	0.82	0.35	0.25	0.92	0.56	0.54
		Protease	3,297	86.40	19.50	1.31	0.82	0.35	0.24	0.90	0.57	0.49
**Phase 5**	*Standard*	Control	3,344	86.50	19.80	1.33	0.82	0.39	0.25	0.92	0.61	0.49
		Protease	3,344	86.40	19.40	1.29	0.86	0.38	0.25	0.91	0.68	0.46
	*Low AA*	Control	3,314	86.30	16.80	1.13	0.71	0.32	0.22	0.78	0.63	0.47
		Protease	3,314	86.90	17.40	1.14	0.75	0.33	0.22	0.80	0.53	0.47
**Phase 6**	*Standard*	Control	3,328	88.10	17.60	1.19	0.76	0.32	0.22	0.76	0.49	0.41
		Protease	3,328	87.70	17.80	1.23	0.77	0.31	0.23	0.78	0.58	0.51
	*Low AA*	Control	3,310	87.20	15.60	1.07	0.67	0.27	0.19	0.69	0.55	0.42
		Protease	3,310	87.90	15.20	1.02	0.62	0.24	0.18	0.69	0.55	0.43
**Phase 7**	*Standard*	Control	3,352	86.70	16.20	0.99	0.62	0.28	0.19	0.74	0.56	0.40
		Protease	3,352	86.70	15.30	1.02	0.63	0.28	0.19	0.68	0.50	0.34
	*Low AA*	Control	3,328	86.50	13.80	0.90	0.55	0.23	0.17	0.63	0.45	0.37
		Protease	3,328	86.90	13.10	0.85	0.54	0.23	0.16	0.61	0.57	0.42
**Phase 8**	*Standard*	Control	3,364	87.40	13.00	0.88	0.55	0.26	0.17	0.61	0.42	0.34
		Protease	3,364	87.30	13.60	0.89	0.55	0.26	0.17	0.62	0.52	0.39
	*Low AA*	Control	3,343	87.50	11.90	0.74	0.49	0.21	0.14	0.56	0.55	0.38
		Protease	3,343	87.00	12.60	0.72	0.49	0.22	0.14	0.57	0.45	0.40
**Phase 9**	*Standard*	Control	3,368	86.40	12.90	0.77	0.53	0.25	0.15	0.60	0.39	0.33
		Protease	3,368	86.90	12.50	0.77	0.53	0.24	0.14	0.57	0.45	0.29
	*Low AA*	Control	3,348	86.90	11.00	0.72	0.49	0.22	0.14	0.56	0.28	0.26
		Protease	3,348	87.00	12.40	0.73	0.51	0.24	0.14	0.55	0.42	0.26

^*a*^Standard: Wean-to-finish feeding program formulated to meet or exceed the NRC (2012) recommended requirements for pigs, including phytase (2,500 FTU/kg in Phase 1 to 4; 500 FTU/kg in Phase 5 to 9); low AA: standard diet with a 15% reduction in SID AA levels. Protease was included at 0.0125% at the expense of corn in standard and low AA diets to create standard + protease and low AA + protease diets (Jefo Protease, Jefo Nutrition Inc., Saint-Hyacinthe, Qc, Canada).

^*b*^Calculated composition.

### Growth Performance and Carcass Characteristics

No interactions were observed between dietary AA supply and protease inclusion on pig growth performance throughout the wean-to-finish period (Table 4). During the first 2 wk postweaning, there were no differences among dietary treatments for overall growth performance. From d 15 postweaning and during most of the wean-to-finish period, pigs fed Standard diets had greater BW, ADG, and G:F (*P* < 0.05) than pigs fed Low AA diets indicating success in formulating a diet limiting in AA. Supplemental protease at 0.0125% inclusion level did not result in improved pig growth performance throughout the wean-to-finish period.

**Table 4.** Main effects of dietary amino acid supply and protease inclusion on pig growth performance throughout the wean-to-finish period^*a*^

	Amino acid supply^*b*^		Protease^*c*^			*P*-value		
Item	Standard	Low AA	0%	0.0125%	SEM	AA supply	Protease	AA supply × protease
Initial BW, kg	5.85	5.85	5.84	5.87	0.080	0.964	0.655	0.910
**D 0–15**								
BW d15, kg	8.16	8.20	8.25	8.12	0.111	0.767	0.212	0.714
ADG, kg/d	0.154	0.156	0.160	0.150	0.0045	0.609	0.062	0.691
ADFI, kg/d	0.225	0.235	0.236	0.224	0.0070	0.139	0.121	0.415
G:F	0.690	0.666	0.685	0.672	0.0199	0.236	0.520	0.170
**D 15–29**								
BW d29, kg	14.52	13.74	14.27	13.10	0.250	0.004	0.288	0.988
ADG, kg/d	0.455	0.396	0.431	0.420	0.0118	<0.0001	0.383	0.934
ADFI, kg/d	0.595	0.576	0.594	0.577	0.0139	0.166	0.234	0.882
G:F	0.765	0.688	0.726	0.727	0.0125	<0.0001	0.303	0.819
**D 29–43**								
BW d43, kg	23.38	21.98	22.86	22.50	0.344	<0.001	0.315	0.909
ADG, kg/d	0.634	0.589	0.614	0.608	0.0100	<0.001	0.572	0.758
ADFI, kg/d	1.038	0.996	1.029	1.006	0.0175	0.023	0.193	0.845
G:F	0.611	0.591	0.597	0.605	0.0076	0.014	0.288	0.979
**D 0–43**								
ADG, kg/d	0.408	0.375	0.396	0.387	0.0066	<0.0001	0.194	0.842
ADFI, kg/d	0.610	0.594	0.611	0.594	0.0103	0.121	0.109	0.938
G:F	0.669	0.632	0.649	0.652	0.0036	<0.0001	0.301	0.366
**D 43–57**								
BW d57, kg	35.2	33.3	34.4	34.0	0.32	<0.0001	0.377	0.998
ADG, kg/d	0.842	0.809	0.827	0.824	0.0069	0.002	0.721	0.767
ADFI, kg/d	1.489	1.463	1.489	1.462	0.0144	0.214	0.192	0.357
G:F	0.566	0.553	0.556	0.564	0.0040	0.030	0.178	0.107
**D 57–85**								
BW d85, kg	63.6	60.7	62.2	62.1	0.46	<0.0001	0.815	0.960
ADG, kg/d	1.016	0.980	0.994	1.003	0.0070	0.001	0.379	0.909
ADFI, kg/d	2.182	2.171	2.164	2.189	0.0194	0.698	0.363	0.764
G:F	0.466	0.452	0.460	0.458	0.0023	<0.0001	0.682	0.792
**D 85–113**								
BW d113, kg	92.7	89.2	91.5	90.4	0.57	<0.0001	0.195	0.838
ADG, kg/d	1.041	1.020	1.047	1.014	0.0076	0.005	0.056	0.512
ADFI, kg/d	2.856	2.806	2.865	2.798	0.0210	0.033	0.105	0.440
G:F	0.364	0.364	0.365	0.363	0.0020	0.471	0.107	0.885
**D 113–134**								
BW d134, kg	115.2	111.2	113.6	112.8	0.68	<0.0001	0.411	0.745
ADG, kg/d	1.071	1.048	1.053	1.066	0.0104	0.133	0.388	0.632
ADFI, kg/d	3.148	3.077	3.098	3.127	0.0219	0.030	0.361	0.519
G:F	0.340	0.341	0.340	0.341	0.0021	0.922	0.730	0.971
**D 43–134**								
ADG, kg/d	1.010	0.982	0.998	0.993	0.0059	0.002	0.557	0.719
ADFI, kg/d	2.506	2.467	2.491	2.481	0.0159	0.093	0.650	0.591
G:F	0.403	0.398	0.401	0.400	0.0012	0.007	0.773	0.674
**D 0–134**								
ADG, kg/d	0.817	0.787	0.805	0.799	0.0048	<0.0001	0.367	0.715
ADFI, kg/d	1.898	1.866	1.888	1.875	0.0125	0.081	0.481	0.661
G:F	0.431	0.422	0.427	0.426	0.0010	<0.0001	0.771	0.764

^*a*^Pigs were assigned to one of the four dietary treatments with nine pens per treatment and 26 pigs per pen.

^*b*^Standard: Wean-to-finish feeding program formulated to meet or exceed the NRC (2012) recommended requirements for pigs, including phytase (2,500 FTU/kg in Phase 1 to 4; 500 FTU/kg in Phase 5 to 9); low AA: standard diet with a 15% reduction in SID AA levels.

^*c*^Protease was included at 0.0125% at the expense of corn in standard and low AA diets to create standard + protease and low AA + protease diets.

**Figure 1.**
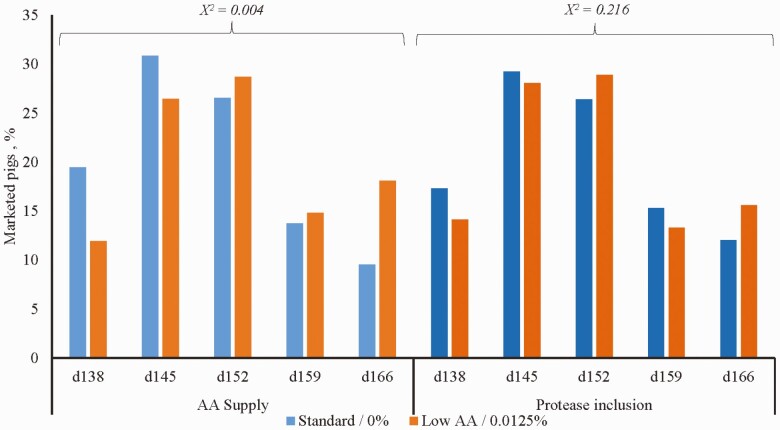
Main effect of dietary amino acid supply and protease supplementation on pig days for market.^1^^1^Standard: Wean-to-finish feeding program formulated to meet or exceed the NRC (2012) recommended requirements for nursery pigs, including superdose level of phytase (2,500 FTU/kg); low AA: standard diet with a 15% reduction in SID AA levels. Protease was included in experimental diets at 0% and 0.0125% at the expense of corn. Data was analyzed as main effects due to no interaction between amino acid supply or protease supplementation levels.

The distribution of pigs selected for shipping over 5 wk of marketing was different between dietary treatments (*χ*^2^ test = 0.004; Figure 1). A greater proportion of pigs fed Standard wean-to-finish diets were shipped in the first 2 wk of marketing; shipments of animals in the last 3 wk consisted primarily of pigs from low AA groups. Furthermore, the means test for this variable indicated differences only on d 138 and d 166, where the Standard group had a greater (*P* < 0.05) percentage of marketed pigs on d 138 and a smaller percentage on d 166. There were no differences among dietary treatments for carcass weight, backfat thickness, loin depth, and FFL (Table 5). However, pigs fed standard diets tended to have greater backfat thickness and loin depth (*P* = 0.06) than pigs fed low AA diets.

**Table 5.** Main effects of dietary amino acid supply and protease inclusion on pig carcass traits^*a*^

	Amino acid supply		Protease			*P*-value		
Item^*b*^	Standard	Low AA	0%	0.0125%	SEM	AA supply	Protease	AA supply × protease
Carcass weight, kg	98.80	98.40	98.52	98.68	0.305	0.330	0.689	0.953
Backfat, mm	19.40	18.18	18.95	18.63	0.496	0.062	0.619	0.298
Loin depth, mm	70.05	67.29	68.31	69.03	1.123	0.061	0.626	0.262
Fat-free lean, %	51.89	52.40	52.04	52.25	0.263	0.197	0.579	0.434

^*a*^Standard: Wean-to-finish feeding program formulated to meet or exceed the NRC (2012) recommended requirements for pigs, including phytase (2,500 FTU/kg in Phase 1 to 4; 500 FTU/kg in Phase 5 to 9); low AA: standard diet with a 15% reduction in SID AA levels. Protease was included at 0.0125% at the expense of corn in standard and low AA diets to create standard + protease and low AA + protease diets. Pigs were marketed over 5 wk according to common industry practice to ensure as many full value pigs as possible.

^*b*^Number of pigs: Carcass Weight (Standard: 405, Low AA: 384, 0% protease: 399, 0.0125% protease: 390); Backfat (standard: 182, low AA: 150, 0% protease: 169, 0.0125% protease: 163); loin depth (standard: 182, low AA: 150, 0% protease: 169, 0.0125% protease: 163); fat-free lean (standard: 182, low AA: 150, 0% protease: 169, 0.0125% protease: 163).

### Intestinal Health

In general, the urinary concentration of lactulose and mannitol decreased from d 5 to d 17 (Table 6). However, urinary L:M ratio was greater on d 17 in comparison with d 5. No statistical difference among dietary treatments was determined for L:M ratio; this measurement was approximately 32% lower in pigs fed low AA + protease compared with standard and low AA pigs at d 5 and d 17 postweaning. Serum concentration of IgA did not differ among dietary treatments at d 5 and d 17 postweaning (Table 6).

**Table 6.** Effect of dietary amino acid supply and protease supplementation on lactulose and mannitol “in urine” and serum IgA concentrations of nursery piglets^*a*^

Item	Standard	Low AA	Low AA + pro	SEM	*P*-value
**Day 5 postweaning**					
Lactulose, mM	2.62	2.51	1.47	0.518	0.242
Mannitol, mM	20.16	21.65	21.66	6.936	0.985
L:M	0.18	0.20	0.13	0.044	0.468
IgA, mg/mL	0.12	0.13	0.14	0.013	0.597
**Day 17 postweaning**					
Lactulose, mM	1.86	1.85	1.52	0.241	0.564
Mannitol, mM	8.13	8.23	8.80	1.366	0.937
L:M	0.28	0.28	0.19	0.042	0.258
IgA, mg/mL	0.47	0.28	0.40	0.071	0.172

^*a*^Standard: Wean-to-finish feeding program formulated to meet or exceed the NRC (2012) recommended requirements for nursery pigs, including superdose level of phytase (2,500 FTU/kg); low AA: standard diet with a 15% reduction in SID AA levels. Protease was included at 0.0125% at the expense of corn in low AA diet to create low AA + pro.

**Figure 2.**
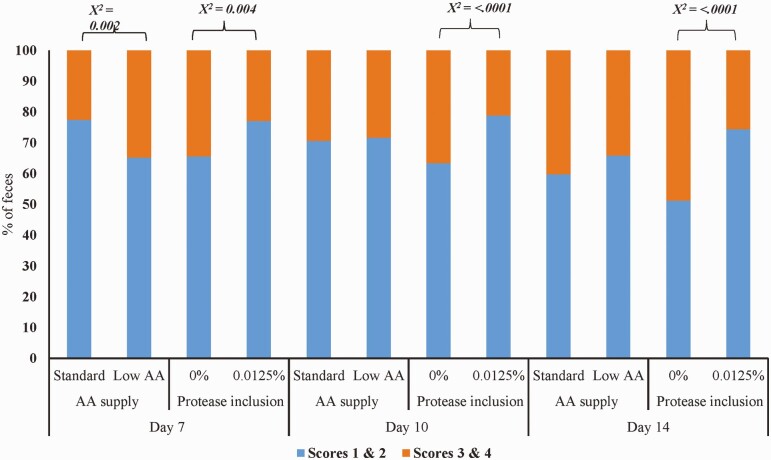
Main effects of dietary amino acid supply and protease inclusion on fecal scores of nursery pigs.^1^^1^Standard: Wean-to-finish feeding program formulated to meet or exceed the NRC (2012) recommended requirements for nursery pigs, including superdose level of phytase (2,500 FTU/kg); low AA: standard diet with a 15% reduction in SID AA levels. Protease was included in experimental diets at 0% and 0.0125% at the expense of corn. Fecal score: scores 1 and 2 represented normal feces and scores of 3 and 4 represented diarrhea. Data was analyzed as main effects due to no interaction between amino acid supply or protease supplementation levels.

On d 7 postweaning, pigs fed low AA diets had more (*χ*^2^ < 0.05) soft and watery feces and, consequently, less normal feces compared with pigs fed standard diets (Figure 2). On d 7, 10, and 14, pigs fed diets with protease supplementation had more (*χ*^2^ < 0.05) normal feces compared with pigs fed diets without protease. No difference was observed among dietary treatment for therapeutic treatment rates during nursery and grow-finish periods (Tables 7).

**Table 7.** Main effect of dietary amino acid supply and protease supplementation on therapeutic antibiotic treatment rate (%) throughout the wean-to-finish period^*a*^

	Amino acid supply		Protease			*P*-value		
Days for market	Standard	Low AA	0%	0.0125%	SEM	AA supply	Protease	AA supply × protease
**Nursery period** ^***b***^								
Treatment rate	1.90	2.09	2.11	1.88	0.186	0.475	0.403	0.615
Respiratory	0.31	0.25	0.26	0.30	0.036	0.197	0.536	0.957
Diarrhea	1.32	1.57	1.58	1.31	0.153	0.260	0.217	0.473
Lame	0.07	0.07	0.11	0.03	0.038	0.153	0.148	0.829
Strep	0.03	0.01	0.02	0.03	0.016	0.372	0.683	0.663
Unthrifty	0.12	0.12	0.10	0.14	0.031	0.922	0.306	0.624
Other	0.04	0.07	0.03	0.08	0.020	0.306	0.141	0.468
**Grow-finish period** ^***b***^								
Treatment rate	0.31	0.24	0.28	0.27	0.033	0.152	0.818	0.115
Respiratory	0.01	0.00	0.01	0.01	0.002	0.103	0.939	0.797
Diarrhea	0.21	0.15	0.17	0.18	0.023	0.072	0.804	0.820
Lame	0.07	0.05	0.08	0.04	0.016	0.407	0.165	0.131
Strep	0.01	0.01	0.01	0.01	0.004	0.885	0.801	0.780
Unthrifty	0.01	0.01	0.01	0.01	0.005	0.408	0.888	0.552
Other	0.01	0.01	0.01	0.01	0.002	0.607	0.323	0.316

^*a*^Standard: Wean-to-finish feeding program formulated to meet or exceed the NRC (2012) recommended requirements for pigs, including phytase (2,500 FTU/kg in Phase 1 to 4; 500 FTU/kg in Phase 5 to 9); low AA: standard diet with a 15% reduction in SID AA levels. Protease was included at 0.0125% at the expense of corn in standard and low AA diets to create standard + protease and low AA + protease diets.

^*b*^Percent of pigs within pen treated with therapeutic antibiotics for respiratory, diarrhea, lameness, strep, un-thriftiness, and, other conditions, respectively.

## DISCUSSION

### Protease Supplementation on Pigs’ Growth Performance and Carcass Characteristics

Dietary protease supplementation can improve N utilization and, consequently, reduce N excretion in manure or ammonia emissions (Tactacan et al., 2016). However, the benefits of dietary supplementation of protease on protein digestibility are not always accompanied by improved growth performance (O’Shea et al., 2014; Upadhaya et al., 2016). The effectiveness of protease in swine diets has been associated with the type of protease used, the dose, feed ingredients used in formulation, and interactions with other enzymes (Cowieson and Roos, 2016; Lee et al., 2018; Torres-Pitarch et al., 2019). In the current study, supplemental protease did not result in improved pig growth performance throughout the wean-to-finish period. This may be related to the dose of protease supplemented to the diets or the inclusion of phytase in all experimental diets. By degrading phytate, phytase also improves AA digestibility through reduction of protein-phytate complexes (Dersjant-Li and Dusel, 2019). Hence, phytase supplementation may act indirectly to improve protein digestion (Lee et al., 2018). The presence of phytase in the diets without protease may explain the lack of response to the protease; these enzymes have been described as possibly not additive (Dos Santos et al., 2017).

To assess the effect of mono-component proteases on performance and apparent ileal digestibility (AID, %) of AA in poultry and swine, Lee et al. (2018) performed a meta-analysis of 67 published trials. The results indicated that the addition of protease in swine diets improved performance (i.e., reduction by 4% of feed conversion ratio) and increased AID values for the majority of AA. However, when other enzymes were included, the beneficial effect of protease on AID of AA was lost, which is in agreement with the results in the present study. Other studies reported beneficial effects of protease supplementation alone (Zuo et al., 2015; Tactacan et al., 2016; Upadhaya et al., 2016) and as part of multi-enzyme complex (Torres-Pitarch et al., 2018; Cowieson et al., 2019; Duarte et al., 2019) on growth performance of pigs.

In the current study, no differences in final carcass weight among dietary treatments were expected due to marketing strategy where animals were marketed over multiple weeks to ensure the maximal number of full-value pigs. The tendency to lower loin depth in pigs fed low AA diets is also expected due to reduction in AA supply. The addition of dietary protease did not cause changes in measured carcass characteristics of pigs, which is in agreement with O’Shea et al. (2014), Choe et al. (2017), Mid et al. (2019), and Lee et al. (2020). This may be related to the lack of effects of dietary protease on growth performance, especially when considering final BW.

### Protease Supplementation in Low Protein Diets

In this experiment, low AA diets were formulated with a 15% reduction in SID AA levels in relation to a standard wean-to-finish feeding program (NRC, 2012). The analyzed composition (Table 3) indicates that the diets were, on average, 12% deficient in lysine content, ranging from 7.4% to 16% through the phases. However, dietary protease supplementation did not offset the reduction in SID AA levels. A reduction of more than 4% to 6% CP in grow-finisher diets affects growth performance and digestive enzymatic production of pigs (He et al., 2016). However, inclusion of exogenous protease in low-protein diets could potentially compensate for the reduction of AA in diets and allow more than 6% dietary CP reduction. In the current study, the reduction in SID AA levels corresponded to a reduction of approximately 9.4% dietary CP. Lei et al. (2017) evaluated effects of protease using diets with high reduction of protein (15.97% vs. 12.94%, which equate to 19% reduction in CP), and reported that supplementation of protease alone in low CP diets improved growth performance and nutrient digestibility of pigs, which is different from the results in this study using protease plus phytase.

Figueroa et al. (2019) assessed the effects of adding protected protease to low AA diets on the growth performance of grow-finisher pigs. Reducing SID lysine content 0.05% and 0.10% relative to the control diet and with a proportionate reduction in concentrations of the remaining AA in the diet, the authors reported no effect of protected protease addition into grow-finish diets on pig growth performance. The benefits of dietary supplementation of protease on protein digestibility are not always accompanied by improved growth performance (O’Shea et al., 2014; Upadhaya et al., 2016). The effectiveness of a protease is associated with the type of protease used, the dose, feed ingredients used in the formulation, and interactions with other enzymes (Cowieson and Roos, 2016; Lee et al., 2018; Torres-Pitarch, et al., 2019). Specifically related to the product and dose used in this study, results from previous work with young pigs where this protease was supplemented at higher dosages (0.020% and 0.030%), showed improvement in growth performance (Zuo et al., 2015; Tactacan et al., 2016), which indicates that greater dosages of the protease may be required.

All diets used in the current study contained phytase throughout the wean-to-finish period. Effects of the two enzymes—protease and phytase—may not be additive, because proteases improve AA digestibility when added alone, but not when added to diets containing phytase or NSPases (Lee et al., 2018) possibly because the other enzymes also affect AA digestibility. In our study, there was no evidence that the low AA diet supplemented with phytase supported better performance to 43 days compared with the same diet supplemented with protease, but the interaction between the two enzymes on the responses observed cannot be ruled out. The possibility that phytase negates the effects of protease on AA digestibility warrants further investigation.

### Protease Supplementation on Postweaning Intestinal Health

The DSAT based on urinary excretion of lactulose and mannitol and serum concentration of IgA can be used as indirect markers of intestinal permeability and gut inflammation of weaned pigs, respectively (Li et al., 2018; Duarte et al., 2019). These assays can be conducted in the live animal at multiple time points, potentially allowing the identification of changes over time. In the current study, DSAT results and serum concentration of IgA were assessed at d 5 and d 17 in only pigs fed standard, low AA, and low AA + protease diets. The selected dietary treatments and time points used for gut health assessment were based on: (1) comparison of positive and negative controls and negative control + protease, (2) first 3 wk postweaning are associated with increased incidence of intestinal disturbances and overall health issues that compromises pig growth performance, and (3) all pigs/pens were feeding experimental diets by d 4 after weaning. To our knowledge, no data is available about the effects of dietary supplementation of protease and phytase on the intestinal permeability of weaned pigs assessed by DSAT under commercial conditions.

The use of lactulose and mannitol as indirect markers of intestinal barrier function considers that lactulose can only traverse the intestinal wall by paracellular pathways, whereas mannitol passes by both paracellular and transcellular routes (Wijtten et al., 2011). Therefore, an increase in the L:M ratio indicates a decrease in the intestinal barrier function. Regardless of dietary treatments in the current study, the L:M ratio increased from d 5 to d 17, which suggests a progressive loss of barrier function from d 5 to d 17 postweaning. These results are supported by increased incidence of soft and watery feces from d 7 to d 14. However, according to Moeser et al. (2007), the most pronounced increase of intestinal permeability of weaned pigs occurs at 24 h postweaning and then gradually improves over the first 2 wk after weaning. Furthermore, Wang et al. (2016) reported that the intestinal barrier damage associated with weaning can be restored to the preweaning levels by d 7 postweaning; however, other factors can influence the recovery process. The results from this experiment do not reflect previous observations and may be related to the water medications provided to the pigs between d 2 and d 7 of entry to barn, which may have postponed the intestinal disturbances mainly reflected on d 14.

When considering the effects of dietary treatments, the observed decreased L:M ratio results suggest that dietary protease supplementation contributes to the improvement of intestinal functionality by minimizing the loss of the intestinal barrier function of pigs during the first weeks postweaning. These results are correlated with the assessment of fecal scores, where pigs fed diets supplemented with protease had lower incidence of soft and watery feces on d 7, 10, and 14 after weaning. Based on the FeedLogic information for this study, all pens had started on Phase 2 experimental diets by d 4 after weaning, which suggests that the effects on intestinal health can be attributed to protease supplementation. Benefits on the intestinal health of weaning piglets have been associated with protease supplementation, including: improvement of gut morphology (Wang et al., 2011; Zuo et al., 2015; Chen et al., 2017; Duarte et al., 2019), reduction of incidence of diarrhea (Wang et al., 2011; Zuo et al., 2015), oxidative stress relief (Chen et al., 2017), and improvement of immune status (Wang et al., 2011), nutrient digestibility (Wang et al., 2011; Tactacan et al., 2016; Chen et al., 2017), and intestinal ecology (Wang et al., 2011). The mechanisms that explain the effects of protease supplementation on intestinal health of pigs are related to microbial composition in the gastrointestinal tract as a result of the dietary nutrient content. Specifically, it has been proposed that variations in the amount of dietary protein that passes may affect microbial composition in the gastrointestinal tract of piglets (Rist et al., 2013). This is related to a greater or lesser protein fermentation in the small and large intestine due to indigestible protein (i.e. N) that provide a substrate for microbes to ferment and proliferate (Wang et al., 2011; Rist et al., 2013; Cao et al., 2016). High fermentation of protein in the gastrointestinal tract was previously related to postweaning diarrhea and with the growth of potentially pathogenic bacteria (Ball and Aherne, 1987; Wellock et al., 2008). Reduction of the total dietary protein can reduce excessive protein fermentation and hence decrease the incidence of postweaning diarrhea (Heo et al., 2008; Wu et al., 2015). Furthermore, feed enzymes can impact the intestinal microbiota and thus benefit the host (Kiarie et al., 2013; Tactacan et al., 2016). In this sense, protease can modulate gut microbial community by reducing undigested substrates (N), reducing enteric pathogens, and hence favoring the intestinal health of pigs. In the current study, both the reduction of dietary protein and use of protease positively affected the intestinal health of pigs as evidenced by the numeric reduced loss of intestinal barrier function and lower incidence of diarrhea. Wang et al. (2011) and Zuo et al. (2015) also reported lower incidence of diarrhea of weaned pigs when protease was supplemented in the diets.

Finally, in relation to the immune response, early weaning stress is associated with poor immunocompetence (McLamb et al., 2013; Pohl et al., 2017), which results from an immature immune system and the interruption of the supply of immunoglobulins and other components from the sow’s milk. This contributes to a weak immunological response to pathogens, which can result in intestinal disorders and diseases (Stokes et al., 2001). In this regard, immunoglobulins, such as IgA, have been used as biomarkers of gastrointestinal functionality (Pietro et al., 2019). Secretory IgA are antibodies produced by the mucosal surfaces, especially in the gastrointestinal tract, and are directly related to inflammatory responses and maintenance of the intestinal epithelial barrier (Mantis et al., 2011). In the current study, dietary supplementation with phytase and protease did not affect the immune status of the pigs as measured by serum concentration of IgA. Duarte et al. (2019) also reported no effects of either xylanase or protease supplementation on the immune status of weaned pigs as measured by serum and mucosal concentrations of TNF-α, IgG, and IgA.

## CONCLUSIONS

This study provides evidence that dietary protease supplementation benefits the intestinal health of nursery pigs. However, the effects on growth performance were not evident in this study when protease was supplemented on top of standard or low AA wean-to-finish diets that also contained 2,500 FTU of phytase from d 1 to 43 post weaning and 500 FTU of phytase in growing-finishing phases. The optimal protease dosage in feeds as well as the possibility that phytase has a similar effect on AA digestibility as protease warrants further research.
